# Injection therapy in knee osteoarthritis: cortisol, hyaluronic acid, PRP, or BMAC (mesenchymal stem cell therapy)?

**DOI:** 10.3389/fmed.2024.1463997

**Published:** 2024-09-27

**Authors:** Christof Pabinger, Georg Stefan Kobinia, Dietmar Dammerer

**Affiliations:** ^1^Institute for Regenerative Medicine (IRM), Graz, Austria; ^2^Austrian Society of Regenerative Medicine (RegMed), Vienna, Austria; ^3^Division of Orthopaedics and Traumatology, University Hospital Krems, Krems, Austria

**Keywords:** arthritis, OA, osteoarthritis, knee, BMA, mesenchymal stem cells, hyaluronic acid, PRP

## Introduction

Osteoarthritis (OA) is the most common chronic joint disease and a leading cause of disability (1). Globally, the number of prevalent OA cases rises exponentially, with knee OA contributing the most to the overall burden (2). This will lead to substantial healthcare expenditures and an unmet demand for orthopedic surgeons in most Organization for Economic Co-operation and Development (OECD) countries (3, 4). Cell-based therapies are being utilized more frequently, and the outcomes of bone marrow aspirate concentrate (BMAC) or “stem cell therapy” demonstrate promising short- to mid-term results. With the use of BMAC, as discussed by Di Matteo and Kon in their study, “The Dilemma of Drink Selection for the Modern Orthopedic Surgeon,” the decision-making process is becoming increasingly complex, even for experienced practitioners. The question now is: “What should I inject into the patients with osteoarthritis (OA) of the knee? Platelet-rich martini or vodka hyaluronic acid?” (5).

Our primary focus in this article will be on critically comparing various treatment options using published data. Second, we will emphasize addressing specific patient needs and share our perspective on personalized medicine for treating knee osteoarthritis. This opinion is informed by over 20 years of experience as the team doctor for the Austrian national soccer team and the Austrian national ski team, applying these insights to the treatment of regular patients.

## Injection therapy

Injection therapy for knee OA is unquestionable, but the question of which type of injection is not easy. The currently used Kellgren–Lawrence (KL) classification for OA dates back to the year 1957 and is not of great help in decision-making (6).

Currently, we have evidence that BMAC injections even have excellent outcomes in patients with severe OA (7), and implants have become so good that the compound annual growth rate in knee arthroplasty in younger patients has exceeded the demand in the elderly for over 15 years (4). The European Society for Sports Traumatology, Knee Surgery, and Arthroscopy (ESSKA) also recommends platelet-rich plasma (PRP) in OA grades I–III (8). Furthermore, the discrepancy between radiological classification and patient-reported symptoms is well known.

The following key points have to be highlighted in our opinion:

- Conservative means (e.g., stretching, insoles, physio, weight reduction, and cycling) are the first choice of therapy and accompany every injection therapy.
- Mechanical errors, such as patellar malalignment, varus/valgus deviations more than 6°, and ligament or meniscal injuries, must be surgically corrected first—regardless of age—since they are significant risk factors for knee osteoarthritis (9–12).
- Total knee arthroplasty (TKA) is still the gold standard for severe osteoarthritis, not responding to conservative means, with 95% good and excellent results at 20 years (13).

## Corticosteroids

Undoubtedly, intra-articular corticosteroid injections offer clinically perceivable pain relief and functional improvement higher than the placebo effect, but these benefits are typically short-term, often diminishing in clinical relevance after 6 weeks in patients with knee OA (14). The effect may vary substantially in different patient groups, and “appropriate patient” selection is important (15). However, it is not possible to predict which patients are most likely to benefit from intra-articular corticosteroid injections (16).

Patients receiving co-treatment with oral duloxetine (serotonin reuptake inhibitor) and CS injections experience considerable improvement in pain and knee function compared to those who receive a CS injection alone (17).

## Hyaluronic acid

A registered study on 15,000 Medicare patients showed that the patients receiving hyaluronic acid (HA) were associated with a longer time to knee arthroplasty (KA) of 8.7 months (8.3–9.1, *p* < 0.001) as compared to patients without HA. Patients with both intra-articular HA and intra-articular CS had an additional 6.3 months (5.5–7.0, *p* < 0.001) to KA over those with only IA HA (18). In a registry study with 182,000 patients, it was evident that with one course of HA, the mean delay time to total knee arthroplasty was 1.4 years (*p* < 0.0001); patients who received ≥5 courses delayed total knee arthroplasty by 3.6 years (*p* < 0.0001) (19). A recent meta-analysis with 943 patients in 10 randomized controlled trials (RCTs) showed that PRP + HA therapy resulted in more pronounced pain and functional improvement in symptomatic KOA patients than HA treatments (20).

## PRP (platelet-rich plasma)

One shot of PRP injection has been shown to reduce joint pain more effectively and for a longer duration, alleviate symptoms, and enhance the daily activities of living and quality of life compared to CS injections (21). In a meta-analysis comparing HA and PRP, intra-articular PRP injections appeared to be more efficacious than HA injections for the treatment of knee OA. PRP demonstrated superior short-term functional recovery (as measured by IKDC, WOMAC, Lequesne, and visual analog scale [VAS] scores) and long-term benefits (in terms of pain relief and function improvement). Additionally, PRP did not increase the risk of adverse events compared to HA (22).

Recent ESSKA guidelines consider PRP injections appropriate for patients aged ≤ 80 years with knee KL 0-III OA grade after failed conservative non-injective or injective treatments. However, PRP is not recommended as a first-line treatment or for patients with KL IV OA grade (8).

## Bone marrow aspirate (concentrate)

One meta-analysis of 16 short-term studies with 875 patients receiving BMAC showed a significant pain reduction (VAS) from the third month onward (23). Another meta-analysis compared HA, PRP, and BMAC at 6 months and found that all led to a significant improvement in function scores when compared to placebo (24). A third meta-analysis of 27 studies and 1,042 patients with a mean 13-month follow-up compared PRP, BMAC, and HA for knee OA: these meta-analyses demonstrated significantly better postinjection WOMAC (*p* < 0.001), VAS (*p* < 0.01), and IKDC scores (*p* < 0.001) in patients who received PRP compared to patients who received HA. Similarly, other meta-analyses demonstrated significantly better postinjection WOMAC (*p* < 0.001), VAS (*p* = 0.03), and IKDC (*p* < 0.001) scores in patients who received BMAC compared to patients who received HA, but no significant differences when comparing PRP and BMAC (25). A further meta-analysis with a mean follow-up of 14 months, comprising 15 studies and 585 patients, found that bone marrow mesenchymal stem cells (BM-MSCs) therapy was most effective in improving VAS and ROM. In contrast, other types of mesenchymal stem cells (MSCs), such as those derived from umbilical cord and adipose tissue, were more effective in improving functional outcomes, including Whole-Organ Magnetic Resonance Imaging Score (WORMS) and Western Ontario McMaster Universities Osteoarthritis Index (WOMAC) scores (26).

To date, no further meta-analyses have been published comparing different sources of pluripotent stem cells or evaluating cell-based injections against established therapeutic options such as HA, CS, and PRP. However, all these meta-analyses have a follow-up of approximately 1 year. However, we know that the positive effect of BMAC is becoming statistically and clinically relevant from the second year onward (7).

Furthermore, a clinical study involving 175 patients directly comparing the outcomes of knee injections with BMAC, PRP, and HA over a 1-year follow-up found that the BMAC group had the best positive effect regarding IKDC and WOMAC at all time points (27). However, another single study reported that the effect of BMAC at 1 year was not superior to that of corticosteroids (28).

To our knowledge, there is only one study with a 4-year follow-up on BMAC therapy in patients with OA. In this study, 35 of 37 knees improved regarding IKDC and WOMAC scores from the first to the last follow-up. IKDC scores significantly increased from 56 ± 12 (range 34–81) to 73 ± 13 (range 45–100), *p* < 0.001. WOMAC scores decreased substantially from 40 ± 23 (range 6–96) to 18 ± 18 (range 0–67), *p* < 0.001 (7).

## Discussion

Undoubtedly, the evolution in injection therapy from CS in 1964 to the current use of PRP and BMAC is impressive. This evidence-based evolution demonstrates that several HA injections can postpone knee arthroplasty by up to 4 years (19). Furthermore, PRP, when combined with HA, can produce even more substantial effects (20).

- **CS injections** are used in acute patients in our institution: for those who need immediate care, for example, days before a tournament, match, or private social event, we use Betamethasone 1 mL.

Regarding HA injections, we switched to the one-treatment 60-mg HA injection a decade ago instead of the 3 times 20 mg to minimize patients' traveling, infection risk, and pain.

- **HA injections** are administered when the characteristic onset of “starting pain” or pain experienced during prolonged sitting with a flexed knee (often referred to as the “cinema sign”) occurs.

We advise symptomatic patients that a renewal of an injection after 6–18 months has a better long-term effect (19). We never inject prophylactically in asymptomatic knees.

Regarding PRP, we currently use a one-treatment system that involves a 60-mL blood withdrawal to produce 4 mL of PRP. This approach seems better to us than the 4–5 times repetitive injections of 1–2 mL PRP, made out of 15 mL because the risk of infection and pain is minimized. The recent ESSKA statement on PRP clarifies its use:

- **PRP injections** are recommended when HA, CS, or conservative treatments are not effective.

Since all meta-analyses on BMAC showed at least equivalent results compared to PRP 1 year after the injection, and considering that the therapeutic effect of BMAC takes 2 years to occur fully, it is most likely that BMAC will outperform PRP in terms of results in the following years (7, 23, 25, 26).

- **BMAC injections** are therefore used in our institution when other injections have failed. We see impressive long-term effects: 35 of 37 patients benefit from this kind of treatment (7). These patient groups are best suited for this treatment:

1. Young patients (< 50 years) who are not yet suitable for knee prosthesis. We know that operating on these patients too early will not result in a “happy patient” (29). Thus, the goal is to gain several years until a prosthesis can be implanted.
2. Old patients (>80 years) with contraindications for surgery or severe comorbidities. Here, the goal is to avoid complications and surgical procedures.
3. “Procrastinating patients” (of all ages) with “no time for a knee prosthesis,” because they have to “take care of relatives,” or are “too much engaged in the jobs at the moment” can also be considered for BMAC injections.

Regarding the injection of intra-articular microfragmented adipose tissue (MF-AT, also known as (mesenchymal) stem cell therapy, stromal vascular fraction (SVF)-therapy), such types of injections have also been proposed for the treatment of knee OA. Several recent studies have shown that a single intra-articular injection of MF-AT is not superior to PRP, meta-analyses on this comparison are currently lacking (30–32). We want to emphasize that our stem cell research study group had significant trouble cultivating fat cells in an experimental setting. In contrast, we could quickly work with bone marrow cells. We, therefore, do not recommend this kind of fat-tissue-derived cell treatment.

We also use all the aforementioned injections in patients with rheumatoid arthritis as an additional option to their basic therapy with a similar outcome as compared to degenerative arthritis. However, the literature on injection therapy in rheumatoid arthritis with autologous PRP or BMA(C) is sparse, and the industry fosters small-molecule and biological therapies, devices, and gene therapy (33).

Based on the aforementioned meta-analyses, we can estimate that future publications will demonstrate the superior long-term effectiveness of BMAC injections as compared to PRP injections (7).

Currently, fluoroscopic guided subchondral injections (of BMAC) for knee osteoarthritis are being discussed, and few pilot study studies show promising results (34, 35). At the moment, a double-blind RCT is conducted, comparing bone marrow aspirate concentrate intra-articular injection combined with subchondral injection vs. intra-articular injection alone for the treatment of symptomatic knee osteoarthritis (36). This might be an option for patients with additional bone marrow edema.

Single studies also recommended the use of BMA instead of BMAC, which makes the procedure faster and cheaper, but this depends on the methodology of the harvesting procedure in order to gain a maximum cell yield (7, 37). This may become an option for the future when the harvesting procedure of BMA becomes standardized.

**Figure 1:**
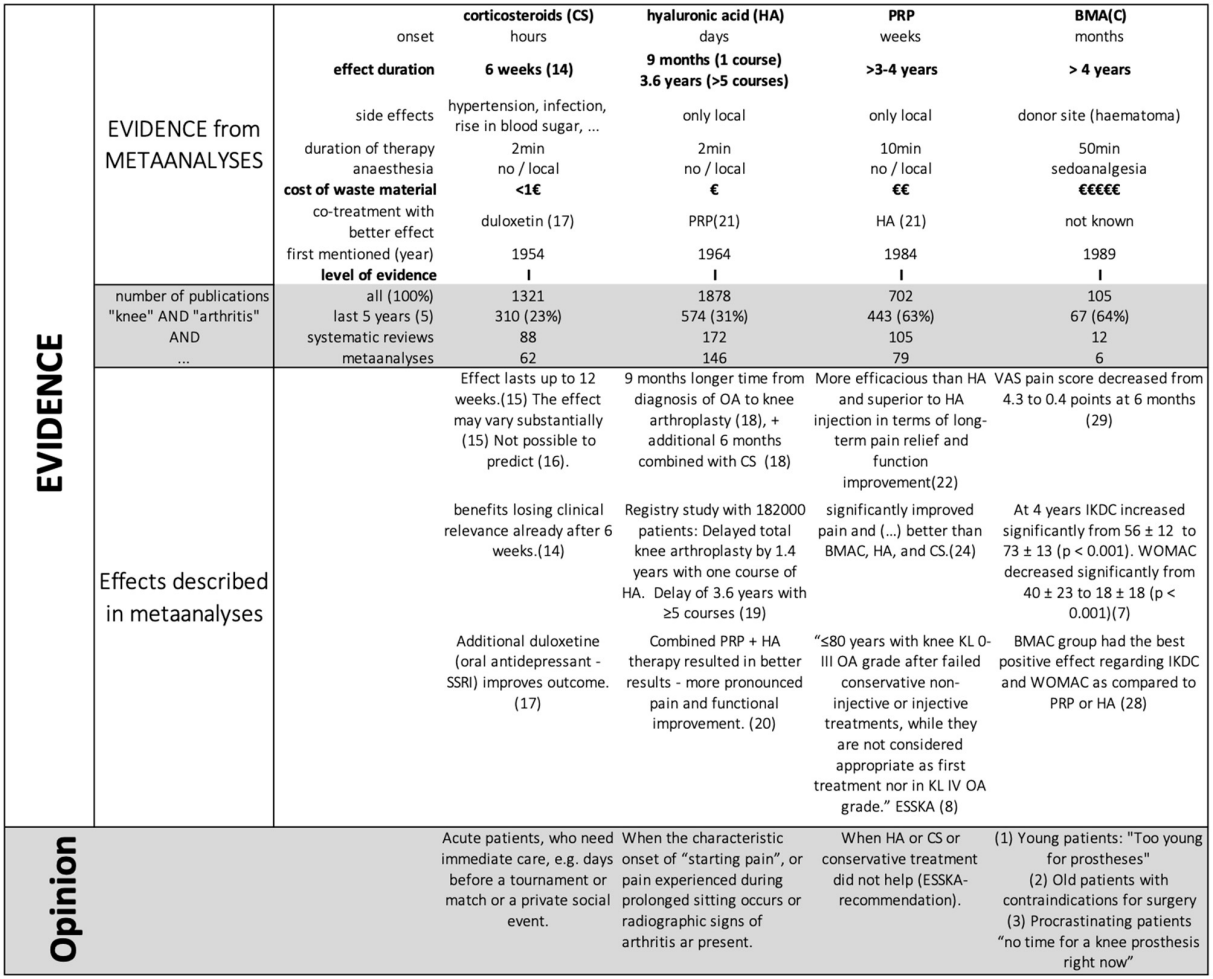
Handout for patients and doctors to choose the appropriate type of injection.

Transferring this evidence into daily clinical praxis, we developed a 1-page handout sheet that can help patients and doctors in choosing the right injection at the right point of time (Figure 1).
